# Developmental Whole Brain White Matter Alterations in Transgenic Huntington’s Disease Monkey

**DOI:** 10.1038/s41598-017-00381-8

**Published:** 2017-03-23

**Authors:** Yuguang Meng, Jie Jiang, Jocelyne Bachevalier, Xiaodong Zhang, Anthony W. S. Chan

**Affiliations:** 1 Yerkes Imaging Center, Yerkes National Primate Research Center, Emory University, Atlanta, GA USA; 2 Department of Human Genetics, Emory University School of Medicine, Atlanta, Georgia USA; 3 Division of Neuropharmacology and Neurologic Diseases, Yerkes National Primate Research Center, Emory University, Atlanta, Georgia USA; 4 Department of Psychology, Emory University School of Medicine, Atlanta, Georgia USA; 5 Division of Developmental and Cognitive Neuroscience, Yerkes National Primate Research Center, Emory University, Atlanta, Georgia USA

## Abstract

Transgenic Huntington’s disease monkey (HD monkey) model provides great opportunity for studying disease progression that could lead to new insight for developing biomarker, early intervention and novel therapeutics. Whole brain white matter integrity of HD-monkeys was examined longitudinally from 6 to 48 months using diffusion tensor imaging (DTI) and tract-based spatial statistics (TBSS). Progressive developmental white matter alterations in HD monkeys were widespread and were observed not only in fiber bundles connecting cortical areas to the striatum (e.g. striatal bundle and external capsule), but also in long association fiber pathways, commissural fibers, and subcortical fiber bundle. In all fiber tracts, the data indicate an arrest in white matter development around 23 months followed by slight decline until adulthood in HD monkeys. The microstructural changes parallel the progressive motor, memory and cognitive decline previously reported as HD monkeys aged. The findings revealed the widespread progressive temporal-spatial microstructural changes in HD monkey brains from infancy to adulthood, suggesting differentiated degenerations across different brain areas during brain development.

## Introduction

Huntington’s disease (HD) is an inherited autosomal dominant neurodegenerative disorder caused by the abnormal expansion of a CAG trinucleotide sequence at the N-terminal of the exon 1 of the Huntingtin (*HTT*) gene located at chromosome 4^1^. HD is characterized by progressive decline in motor function, cognition and psychiatric symptoms^2^. Death is expected in 10 to 20 years after diagnosis primarily based on motor functions^3^. Neurological and behavioral assessment is the primary approach to determine disease progression. Recent development in non-invasive neuroimaging techniques has provided a powerful diagnostic platform to evaluate neuroanatomical and neurochemical changes that may precede the appearance of clinical symptoms^4, 5^.

Multiple longitudinal studies on HD patients are ongoing with a large cohort of participants that aimed to investigate disease onset and progression using unified clinical measurements^6, 7^. Although human longitudinal studies are important for understanding the etiology of HD, disease progression is a relatively slow process and may take decades to evolve from prodromal to clinically manifested stage. Therefore, the development of an animal model with similar genetic constitution, progressive decline in measurable clinical features and neuroanatomical structures, is important for studying HD pathogenesis, the development of biomarkers and novel therapeutics^8, 9^. Firstly, although rodent HD models mimic some clinical features of HD including neuropathology, these models have inherent limitations, such as brain size, neuroanatomy, neural circuitry as well as emotional response^10^. Secondly, rodent early brain development differs greatly from primates, with greater immaturity at birth but more compressed maturation until adulthood, resulting in greater vulnerability that is not seen in humans, and/or exaggerate effects of genetic manipulation^11, 12^. In contrast, nonhuman primates are similar to humans in anatomical structures and functional organizations and in their ontogenetic development^13–15^. Specifically, white matter fiber connections between striatum and prefrontal cortex or motor domains in primates are well established^16^. The use of the HD monkey model allows us to study in the same animals the developmental trajectories of cognitive deficits^8, 17^, motor changes^8^, emotional and hormonal alterations^18^, and the atrophy in specific neural system, such as the striatum, where mutant HTT aggregates were previously reported^8^. Thus, the full spectrum of HD patient symptoms could be modeled in nonhuman primates across the lifespan and be beneficial for the identification of early markers of HD disease and treatment.

Since micro-structural changes are expected to precede macro-structural changes, comparison between macro-structural imaging and micro-structural imaging could provide insight on progressive neurobiological changes^4^. Diffusion tensor imaging (DTI) is a non-invasive MRI technique for investigating micro-structural integrity of neuronal fibers^19^. Fractional anisotropy (FA), a scalar measure of the degree of anisotropic water diffusion of brain tissues, is commonly used for quantitative assessment of changes in white matter. Additionally, mean diffusivity (MD) characterizes the overall displacement of water molecules in tissue and is also related to the microstructural features of white matter organization^20^. In particular, axial diffusivity (D_a_) and radial diffusivity (D_r_) can be used to reveal neuropathological changes, such as the disruption and loss of axonal membranes and myelin in the fiber tracts as well as changes in size, density and organization of axons^21, 22^. In addition, changes of microglia cells, astrocytosis, neuronal remodeling, or loss of specific fiber tracts could change FA and diffusivities in brain structures^23, 24^.

Although prior studies have suggested that DTI measurement is a valuable bio-marker to assess HD severity in human, limited longitudinal study has been performed to evaluate progressive changes^25–31^. In this study, DTI was performed on HD monkeys and age-matched control monkeys starting at six-months and at every 6-months interval up to 48 months of age to assess changes in the integrity of the white matter across development. To perform exploratory unbiased analysis without any prior assumptions, tract-based spatial statistics (TBSS), a robust and sensitive approach for voxelwise multiple-subject comparisons of DTI data, was utilized to study white matter in fiber tracts and brain structures in this work^32^. Since rhesus monkeys have shorter life-span than humans^33^ and develop progressive changes in various clinical measurements^8, 17^, HD monkeys could be an ideal preclinical animal model for efficient assessments of early disease markers and novel therapeutics.

## Results

**Figure 1:**
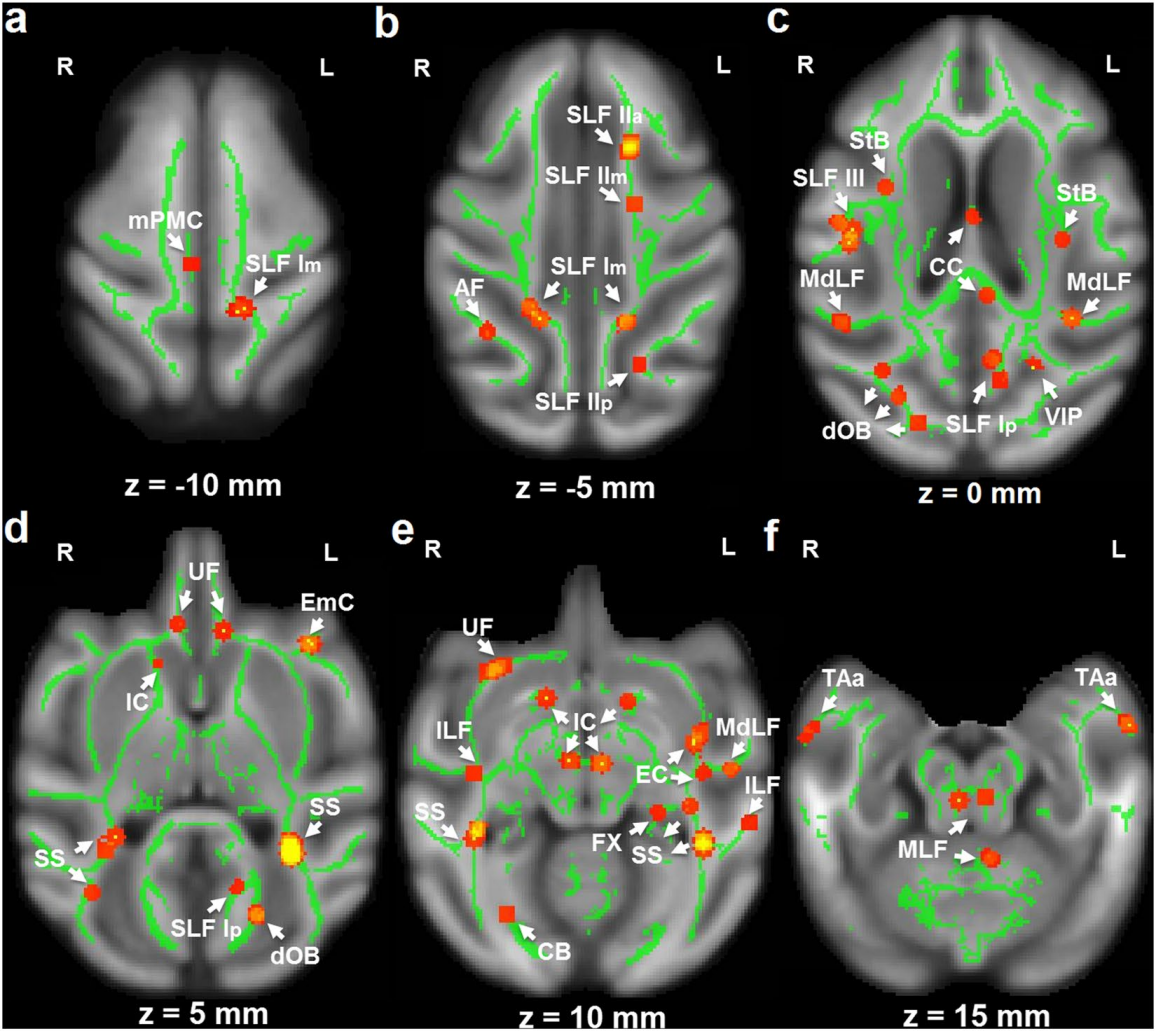
TBSS analysis of the whole brain through the horizontal direction (z). Areas with red-yellow color (scale: from low to high) indicate significant group difference for the ages with maximum FA (p < 0.05, corrected with FDR). To facilitate visualization, voxels showing significant difference were thickened using the “tbss_fill” script implemented in the FSL software. In the following ROI-based analysis, DTI-derived measures within the areas were averaged from the corresponding skeletonized map (e.g., FA, in green color). L, R: left or right hemisphere.

Comparisons of the age at which maximal FA value was reached for the controls and HD animals indicated significant age differences in several brain structures including both gray and white matter (see Region of Interests (ROIs) in Fig. 1). These ROIs were selected for further analyses (see below) and included cortical areas, such as the right medial primary motor cortex (mPMC), the left ventral intraparietal cortex (VIP), and the anterior temporal area (TAa) bilaterally. ROIs in white matter included (a) long association fiber pathways, i.e. all three subcomponents of the superior longitudinal fasciculus (SLF) coursing between the parietal and frontal lobes (middle and posterior parts of SLF subcomponent I (i.e., SLF Im, SLF Ip)); anterior, middle and posterior parts of the SLF subcomponent II (i.e. SLF IIa, SLF IIm, SLF IIp); and middle part of SLF subcomponent III, the posterior part of arcuate fasciculus (AF) coursing from the parietotemporal region to the frontal lobe, the anterior part of the extreme capsule (EmC) coursing from the superior temporal gyrus to the frontal lobe, the middle and posterior parts of middle longitudinal fasciculus (MdLF) coursing from the parietal cortex to the temporal pole, the uncinate fasciculus (UF) coursing from the anterior temporal lobe to the medial and orbital prefrontal cortex, the occipital part of cingulum bundle (CB), parietal and mid-temporal parts of the inferior longitudinal fasciculus (ILF), and the dorsal occipital bundle (dOB) coursing from the preoccipital region to the calcarine. Other ROIs included the striatal bundles (StB) and external capsule (EC), the commissural fibers, i.e. the corpus callosum (anterior part of CC), the projection fibers, i.e., the internal capsule (IC) traveling to the brain stem and the sagittal stratum (SS) including fibers from the occipital cortex to the lateral geniculate nucleus and the medial longitudinal fasciculus (MLF), and the fornix (FX) coursing from the hippocampus to the anterior thalamus and ventromedial prefrontal cortex.

**Figure 2:**
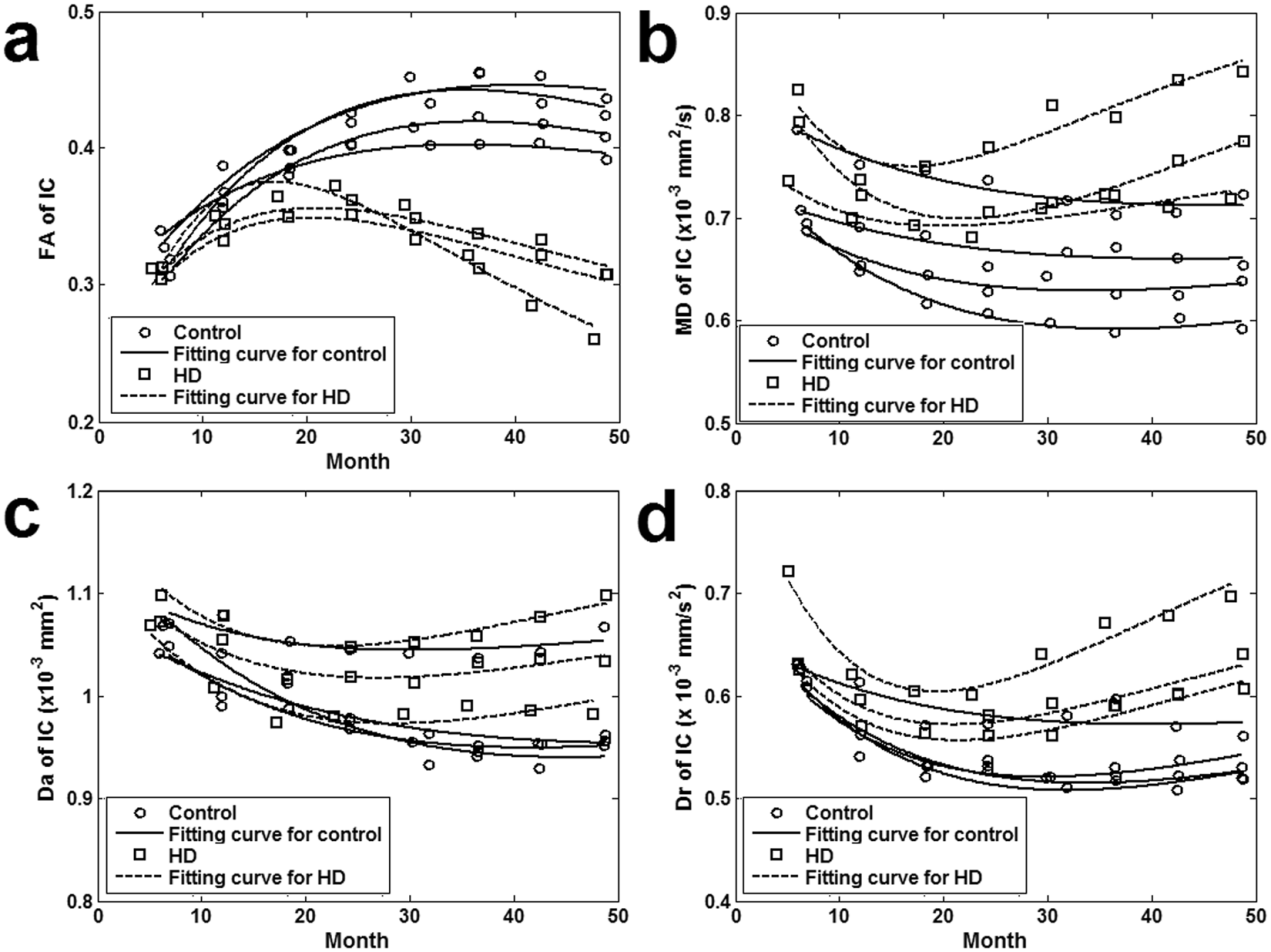
An illustration of the developmental changes of the DTI-derived measures across ages for the internal capsule (IC) of HD and control animals, fitted with the Poisson model. FA: fractional anisotropy; MD: mean diffusivity; Da: axial diffusivity; Dr: radial diffusivity.

**Figure 3:**
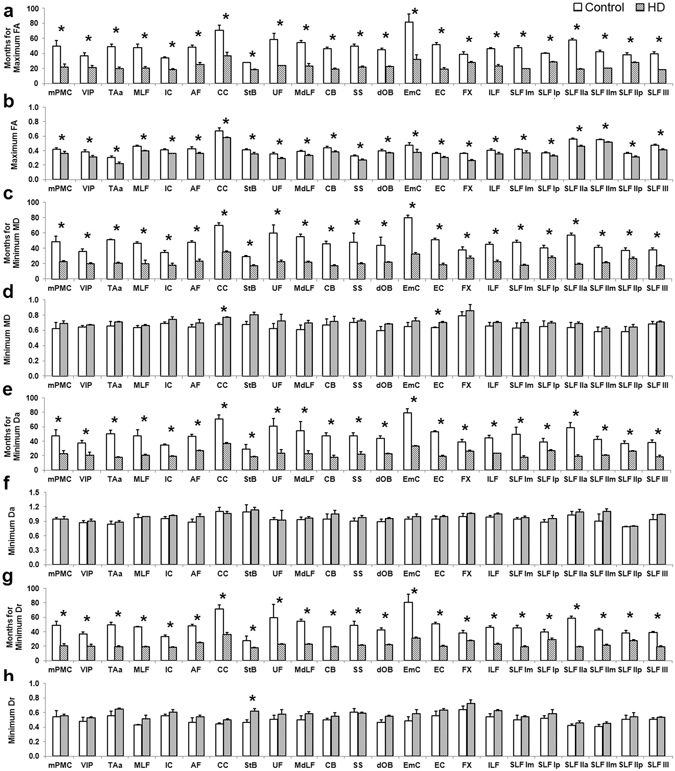
A graphical representation of the age (months) for the minimum/maximum DTI-derived measures (**a**,**c**,**e**,**g**), and for the minimum/maximum DTI-derived measures (**d**,**f**,**h**; unit: 10^−3^ mm^2^/s) averaged across all ages for each ROI. Details are shown in Tables 1 and 2. The star (*) indicates significant group difference with FDR correction (q = 0.05). FA: fractional anisotropy; MD: mean diffusivity; Da: axial diffusivity; Dr: radial diffusivity. Note that for Fig. 3(a,c,e,g), the y-axis value represents the age (months) predicted by the Poisson model and thus could exceed the actual age range of the animals used in the study (48 months).

**Figure 4:**
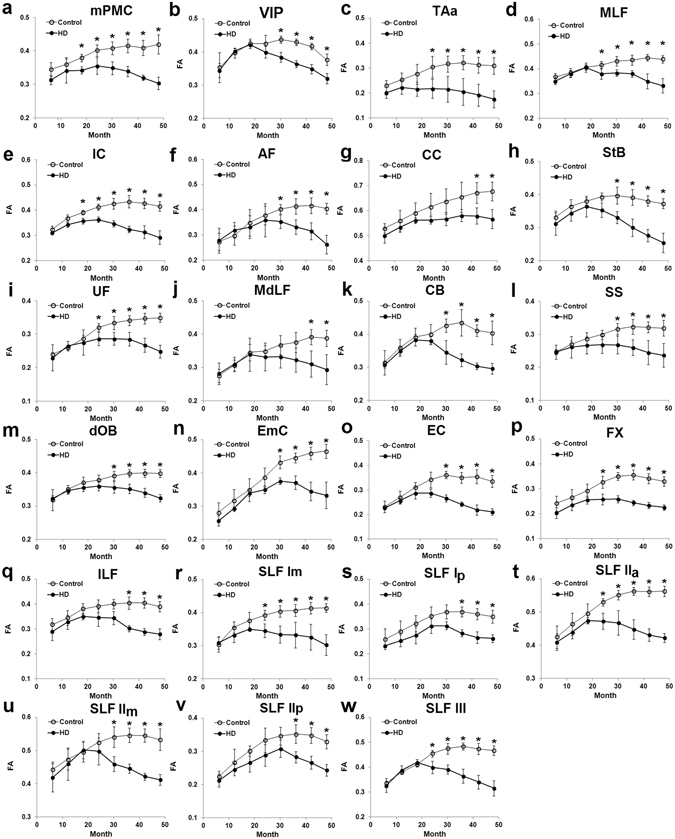
The averaged FA values in all ROIs at each age for both groups as determined by TBSS. The star (*) shows significant group difference at the specific age (*p < 0.05; details are shown in Tables 3 and 4). FA: fractional anisotropy.

Taking IC as an example, DTI-derived measures of both controls and HD monkeys were fitted across ages using the Poisson model (Fig. 2). The fitting curves revealed that HD animals reached peak (FA)/bottom (MD) DTI-measures at younger ages than controls. HD monkeys also reached lower peak FA value and higher diffusivity measures (i.e., MD, Da and Dr) than control monkeys at 34 months of age. Further analyses indicated that the maximal FA value was reached at a younger age for the HD monkeys (22.7 ± 4.8 months) than for the controls (47.8 ± 11.7), revealing an arrest of white matter maturation in the HD group predicted by Poisson model (Fig. 3a,c,e and g; see Table 1 for details). Across ages, HD monkeys had significantly lower maximal FA values in all ROIs (Fig. 3b), but significantly higher minimum MD values only in CC and EC (Fig. 3d) as compared to controls. Finally, as compared to controls, the minimum Dr values of HD monkeys were significantly higher only in the fibers of StB (Fig. 3h), whereas the minimum Da values showed no difference between the two groups in all areas (Fig. 3f; see Table 2 for details). A two-way ANOVA analysis for FA values indicated a significant interaction between age and group for all ROIs (all ps < 0.05; see Fig. 4). Post hoc analysis (Tables 3 and 4) showed that no significant FA changes were observed between the two groups in any ROI at the early ages of 6 and 12 months. By 18 months of age, only mPMC and IC showed significant group differences in FA values (p < 0.05) and beginning at 24 months of age, more areas (MLF, UF, TAa, FX, SLF I, SLF II, SLF III) showed significant group differences in FA values. Finally by 42 months, significant group differences in FA values were observed in all ROIs. These results suggest that the emergence of white matter alterations for HD animals varied according to the different areas.

**Table 1:** Group difference in age for the minimum/maximum DTI-derived measures based on analysis of the Poisson model and TBSS.

Area	Age for Maximum FA (month) (Mean ± SD)			Age for Minimum MD (month) (Mean ± SD)			Age for Minimum Da (month) (Mean ± SD)			Age for Minimum Dr (month) (Mean ± SD)		
	Control	HD	p	Control	HD	p	Control	HD	p	Control	HD	p
mPMC	49.4 ± 7.8	21.6 ± 4.2	**0.002**	48.3 ± 7.7	22.8 ± 0.8	**0.003**	47.4 ± 8.5	22.1 ± 5.0	**0.006**	48.8 ± 5.7	20.4 ± 3.0	**0.001**
VIP	36.7 ± 4.4	20.9 ± 3.1	**0.002**	35.7 ± 3.6	20.3 ± 1.0	**0.001**	37.8 ± 3.5	20.5 ± 4.1	**0.001**	36.8 ± 3.0	19.9 ± 2.6	**0.001**
TAa	49.1 ± 3.4	19.8 ± 2.2	<**0.001**	51.2 ± 0.8	20.8 ± 1.1	<**0.001**	50.5 ± 4.8	17.4 ± 0.8	<**0.001**	49.9 ± 2.9	19.1 ± 1.9	<**0.001**
MLF	47.5 ± 4.5	19.9 ± 2.4	<**0.001**	46.5 ± 2.2	19.9 ± 4.3	<**0.001**	47.7 ± 8.0	20.5 ± 1.8	**0.002**	46.7 ± 0.8	18.9 ± 0.9	<**0.001**
IC	34.0 ± 1.2	18.5 ± 1.0	<**0.001**	34.6 ± 2.5	18.4 ± 2.3	<**0.001**	34.8 ± 1.7	19.2 ± 0.6	<**0.001**	33.6 ± 1.7	18.5 ± 0.5	<**0.001**
AF	48.3 ± 2.9	24.8 ± 3.1	<**0.001**	47.7 ± 2.1	23.8 ± 2.4	<**0.001**	47.0 ± 2.4	26.7 ± 1.1	<**0.001**	48.2 ± 2.3	24.3 ± 1.6	<**0.001**
CC	70.4 ± 7.2	36.4 ± 5.2	**0.001**	69.8 ± 3.3	35.5 ± 1.2	<**0.001**	70.6 ± 5.8	36.4 ± 1.7	<**0.001**	71.4 ± 5.9	35.9 ± 3.0	<**0.001**
StB	27.6 ± 0.5	18.2 ± 1.0	**0.001**	28.9 ± 1.4	17.6 ± 1.0	<**0.001**	29.3 ± 5.8	18.5 ± 0.6	**0.03**	27.8 ± 6.1	17.7 ± 0.8	**0.04**
UF	58.2 ± 7.9	23.3 ± 0.6	**0.001**	59.9 ± 10.6	22.6 ± 2.1	**0.002**	61.1 ± 10.6	23.1 ± 5.3	**0.003**	59.6 ± 18.0	22.1 ± 1.6	**0.02**
MdLF	54.3 ± 2.9	23.2 ± 3.3	<**0.001**	55.3 ± 3.4	22.2 ± 0.7	<**0.001**	54.6 ± 12.5	22.5 ± 4.2	**0.009**	54.6 ± 2.5	22.2 ± 1.4	<**0.001**
CB	46.5 ± 1.9	18.7 ± 2.0	<**0.001**	46.1 ± 2.8	17.4 ± 0.9	<**0.001**	47.3 ± 4.4	17.5 ± 3.0	<**0.001**	45.5 ± 0.5	18.6 ± 1.7	<**0.001**
SS	49.4 ± 3.2	21.5 ± 1.8	<**0.001**	48.0 ± 11.8	20.5 ± 0.9	**0.009**	47.4 ± 4.4	21.4 ± 4.4	**0.001**	48.8 ± 5.6	20.9 ± 0.8	<**0.001**
dOB	44.5 ± 2.5	22.5 ± 1.0	<**0.001**	43.9 ± 10.3	21.9 ± 0.5	**0.02**	43.6 ± 3.7	22.5 ± 1.1	<**0.001**	42.3 ± 3.0	21.7 ± 0.9	<**0.001**
EmC	81.5 ± 10.6	32.0 ± 6.1	**0.001**	79.6 ± 3.6	32.6 ± 1.9	<**0.001**	79.0 ± 6.0	33.1 ± 0.6	<**0.001**	80.3 ± 11.4	31.1 ± 1.5	**0.001**
EC	51.4 ± 2.7	19.1 ± 2.3	<**0.001**	51.0 ± 2.2	19.0 ± 1.6	<**0.001**	52.7 ± 1.5	18.6 ± 2.3	<**0.001**	50.9 ± 2.5	19.6 ± 2.1	<**0.001**
FX	38.9 ± 3.2	27.7 ± 1.8	**0.002**	37.7 ± 3.8	27.5 ± 2.2	**0.001**	39.0 ± 3.7	26.3 ± 1.5	**0.003**	38.5 ± 3.0	27.4 ± 1.2	**0.002**
ILF	45.9 ± 1.4	23.0 ± 2.0	<**0.001**	45.2 ± 2.6	22.8 ± 1.7	<**0.001**	44.7 ± 3.1	23.0 ± 0.7	<**0.001**	46.1 ± 2.2	22.6 ± 1.7	<**0.001**
SLF Im	47.3 ± 3.0	19.2 ± 0.5	<**0.001**	47.8 ± 2.6	18.2 ± 1.1	<**0.001**	49.6 ± 9.7	17.8 ± 1.9	**0.003**	45.2 ± 3.7	18.9 ± 1.5	<**0.001**
SLF Ip	39.9 ± 1.0	28.4 ± 0.9	<**0.001**	40.2 ± 3.8	28.4 ± 1.7	<**0.001**	38.6 ± 5.6	26.7 ± 1.9	**0.02**	39.7 ± 3.4	29.0 ± 1.9	**0.005**
SLF IIa	57.8 ± 2.0	18.7 ± 0.8	<**0.001**	57.2 ± 2.7	19.8 ± 1.1	<**0.001**	58.9 ± 7.2	18.9 ± 2.4	<**0.001**	58.4 ± 3.0	19.1 ± 0.9	<**0.001**
SLF IIm	42.3 ± 1.7	20.0 ± 0.8	<**0.001**	41.3 ± 2.2	21.4 ± 1.2	<**0.001**	42.8 ± 3.8	20.6 ± 1.0	<**0.001**	42.2 ± 2.3	21.0 ± 1.9	<**0.001**
SLF IIp	38.1 ± 2.7	27.5 ± 1.3	**0.001**	37.0 ± 3.6	26.8 ± 1.5	**0.001**	37.0 ± 3.2	25.9 ± 0.8	**0.002**	38.6 ± 3.2	27.7 ± 1.1	**0.003**
SLF III	39.5 ± 2.6	18.2 ± 0.6	<**0.001**	37.9 ± 2.8	17.3 ± 1.3	**0.03**	38.4 ± 3.5	17.9 ± 3.0	<**0.001**	38.9 ± 1.4	18.9 ± 1.8	**0.001**

These data were tested as significant group difference with independent t-test using FDR correction with q value 0.05. SD: standard derivation. FA: fractional anisotropy; MD: mean diffusivity; Da: axial diffusivity; Dr: Radial diffusivity.

**Table 2:** Group difference in the minimum/maximum DTI-derived measures based on analysis of the Poisson model and TBSS.

Area	FA (Mean ± SD)			MD (×10^−3^ mm^2^/s) (Mean ± SD)			Da (×10^−3^ mm^2^/s) (Mean ± SD)			Dr (×10^−3^ mm^2^/s) (Mean ± SD)		
	Control	HD	p	Control	HD	p	Control	HD	p	Control	HD	p
mPMC	0.42 ± 0.03	0.36 ± 0.04	0.04*	0.62 ± 0.08	0.68 ± 0.04	0.28	0.94 ± 0.04	0.94 ± 0.05	0.99	0.54 ± 0.08	0.56 ± 0.02	0.79
VIP	0.46 ± 0.01	0.41 ± 0.02	0.008*	0.64 ± 0.02	0.66 ± 0.01	0.17	0.87 ± 0.04	0.90 ± 0.04	0.34	0.48 ± 0.05	0.52 ± 0.02	0.22
TAa	0.31 ± 0.02	0.22 ± 0.02	0.002*	0.65 ± 0.06	0.70 ± 0.02	0.25	0.84 ± 0.06	0.88 ± 0.04	0.36	0.55 ± 0.07	0.64 ± 0.02	0.09
MLF	0.46 ± 0.01	0.39 ± 0.02	0.002*	0.64 ± 0.03	0.66 ± 0.01	0.21	0.98 ± 0.07	0.99 ± 0.01	0.79	0.43 ± 0.01	0.51 ± 0.05	0.01
IC	0.41 ± 0.01	0.36 ± 0.01	0.001*	0.69 ± 0.02	0.74 ± 0.04	0.08	0.95 ± 0.05	1.01 ± 0.01	0.09	0.56 ± 0.02	0.60 ± 0.03	0.07
AF	0.42 ± 0.03	0.36 ± 0.02	0.02*	0.64 ± 0.03	0.69 ± 0.05	0.15	0.89 ± 0.06	0.99 ± 0.06	0.07	0.47 ± 0.06	0.54 ± 0.03	0.11
CC	0.67 ± 0.04	0.58 ± 0.01	0.02*	0.68 ± 0.02	0.77 ± 0.01	0.001*	1.11 ± 0.09	1.06 ± 0.04	0.43	0.44 ± 0.01	0.49 ± 0.02	0.01
StB	0.41 ± 0.02	0.35 ± 0.02	0.01*	0.68 ± 0.04	0.80 ± 0.04	0.008	1.10 ± 0.14	1.13 ± 0.05	0.68	0.47 ± 0.03	0.62 ± 0.04	0.002*
UF	0.35 ± 0.02	0.29 ± 0.02	0.007*	0.62 ± 0.06	0.72 ± 0.09	0.14	0.94 ± 0.03	0.92 ± 0.20	0.87	0.51 ± 0.06	0.58 ± 0.06	0.20
MdLF	0.39 ± 0.01	0.33 ± 0.02	0.004*	0.61 ± 0.06	0.69 ± 0.04	0.10	0.93 ± 0.06	0.96 ± 0.04	0.56	0.50 ± 0.06	0.58 ± 0.03	0.08
CB	0.44 ± 0.02	0.38 ± 0.01	0.01*	0.67 ± 0.08	0.71 ± 0.07	0.42	0.94 ± 0.10	1.04 ± 0.08	0.22	0.50 ± 0.02	0.55 ± 0.05	0.16
SS	0.32 ± 0.02	0.26 ± 0.02	0.01*	0.70 ± 0.05	0.72 ± 0.02	0.73	0.90 ± 0.07	0.98 ± 0.04	0.17	0.60 ± 0.05	0.59 ± 0.02	0.64
dOB	0.40 ± 0.02	0.36 ± 0.01	0.02*	0.59 ± 0.06	0.68 ± 0.01	0.06	0.89 ± 0.05	0.95 ± 0.02	0.11	0.46 ± 0.04	0.55 ± 0.01	0.02
EmC	0.47 ± 0.03	0.37 ± 0.04	0.02*	0.65 ± 0.05	0.72 ± 0.04	0.11	0.94 ± 0.05	1.00 ± 0.05	0.24	0.49 ± 0.06	0.58 ± 0.06	0.09
EC	0.36 ± 0.02	0.30 ± 0.01	0.007*	0.63 ± 0.01	0.70 ± 0.02	0.001*	0.94 ± 0.07	0.99 ± 0.02	0.27	0.56 ± 0.06	0.63 ± 0.03	0.13
FX	0.36 ± 0.01	0.26 ± 0.02	0.001*	0.79 ± 0.06	0.85 ± 0.08	0.29	0.99 ± 0.07	1.06 ± 0.02	0.15	0.64 ± 0.04	0.72 ± 0.05	0.09
ILF	0.41 ± 0.02	0.35 ± 0.02	0.02*	0.65 ± 0.05	0.70 ± 0.02	0.19	0.98 ± 0.04	1.04 ± 0.03	0.07	0.54 ± 0.04	0.62 ± 0.02	0.02
SLF Im	0.42 ± 0.01	0.36 ± 0.03	0.03*	0.63 ± 0.07	0.70 ± 0.04	0.17	0.94 ± 0.04	0.97 ± 0.04	0.44	0.50 ± 0.06	0.54 ± 0.02	0.38
SLF Ip	0.37 ± 0.01	0.32 ± 0.02	0.02*	0.65 ± 0.08	0.69 ± 0.02	0.39	0.88 ± 0.05	0.95 ± 0.07	0.17	0.52 ± 0.04	0.58 ± 0.06	0.16
SLF IIa	0.56 ± 0.01	0.46 ± 0.01	<0.001*	0.63 ± 0.07	0.68 ± 0.03	0.28	1.03 ± 0.07	1.09 ± 0.06	0.34	0.42 ± 0.02	0.46 ± 0.03	0.13
SLF IIm	0.55 ± 0.01	0.51 ± 0.01	0.001*	0.58 ± 0.06	0.63 ± 0.02	0.31	0.90 ± 0.15	1.10 ± 0.06	0.11	0.41 ± 0.02	0.45 ± 0.02	0.07
SLF IIp	0.36 ± 0.02	0.31 ± 0.02	0.01*	0.58 ± 0.07	0.64 ± 0.04	0.28	0.78 ± 0.01	0.79 ± 0.02	0.70	0.51 ± 0.05	0.54 ± 0.06	0.54
SLF III	0.48 ± 0.01	0.41 ± 0.02	0.002*	0.68 ± 0.03	0.70 ± 0.02	0.30	0.93 ± 0.11	1.04 ± 0.01	0.18	0.51 ± 0.02	0.53 ± 0.01	0.11

These data were tested as significant group difference with independent t-test using FDR correction with q value 0.05 (*). SD: standard derivation. FA: fractional anisotropy; MD: mean diffusivity; Da: axial diffusivity; Dr: Radial diffusivity.

**Table 3:** FA group difference at ages from 6 to 24 months.

Area	FA at 6 months (Mean ± SD)			FA at 12 months (Mean ± SD)			FA at 18 months (Mean ± SD)			FA at 24 months (Mean ± SD)		
	Control	HD	p	Control	HD	p	Control	HD	p	Control	HD	p
mPMC	0.34 ± 0.02	0.31 ± 0.01	0.09	0.36 ± 0.02	0.34 ± 0.04	0.13	0.38 ± 0.01	0.34 ± 0.01	0.03*	0.40 ± 0.02	0.35 ± 0.02	0.01*
VIP	0.35 ± 0.04	0.34 ± 0.02	0.66	0.40 ± 0.01	0.40 ± 0.01	0.60	0.42 ± 0.01	0.42 ± 0.01	0.86	0.42 ± 0.03	0.40 ± 0.02	0.45
TAa	0.23 ± 0.02	0.20 ± 0.02	0.14	0.25 ± 0.03	0.22 ± 0.03	0.21	0.28 ± 0.04	0.22 ± 0.03	0.14	0.30 ± 0.04	0.22 ± 0.04	0.04*
MLF	0.37 ± 0.01	0.35 ± 0.02	0.47	0.38 ± 0.02	0.38 ± 0.01	0.75	0.41 ± 0.01	0.41 ± 0.02	0.68	0.42 ± 0.01	0.38 ± 0.02	0.03*
IC	0.32 ± 0.01	0.31 ± 0.01	0.23	0.37 ± 0.01	0.34 ± 0.01	0.08	0.39 ± 0.01	0.36 ± 0.01	0.02*	0.41 ± 0.01	0.36 ± 0.01	0.01*
AF	0.27 ± 0.03	0.28 ± 0.05	0.85	0.30 ± 0.04	0.32 ± 0.03	0.83	0.35 ± 0.05	0.33 ± 0.05	0.71	0.38 ± 0.04	0.36 ± 0.07	0.15
CC	0.53 ± 0.02	0.50 ± 0.03	0.38	0.56 ± 0.04	0.53 ± 0.02	0.62	0.59 ± 0.04	0.56 ± 0.01	0.41	0.62 ± 0.05	0.56 ± 0.02	0.18
StB	0.33 ± 0.02	0.31 ± 0.03	0.54	0.36 ± 0.02	0.34 ± 0.02	0.71	0.38 ± 0.01	0.36 ± 0.03	0.41	0.39 ± 0.01	0.35 ± 0.02	0.05
UF	0.24 ± 0.01	0.23 ± 0.04	0.39	0.26 ± 0.01	0.26 ± 0.01	0.92	0.29 ± 0.01	0.28 ± 0.04	0.55	0.32 ± 0.01	0.29 ± 0.02	0.01*
MdLF	0.28 ± 0.02	0.28 ± 0.03	0.88	0.31 ± 0.02	0.31 ± 0.02	0.93	0.35 ± 0.02	0.34 ± 0.05	0.77	0.35 ± 0.02	0.33 ± 0.04	0.28
CB	0.31 ± 0.04	0.31 ± 0.01	0.87	0.36 ± 0.03	0.35 ± 0.01	0.49	0.39 ± 0.03	0.38 ± 0.02	0.56	0.40 ± 0.03	0.38 ± 0.01	0.33
SS	0.25 ± 0.01	0.25 ± 0.02	0.28	0.27 ± 0.01	0.26 ± 0.03	0.40	0.29 ± 0.02	0.27 ± 0.03	0.34	0.30 ± 0.01	0.27 ± 0.03	0.05
dOB	0.32 ± 0.03	0.32 ± 0.01	0.92	0.35 ± 0.01	0.35 ± 0.02	0.49	0.37 ± 0.02	0.36 ± 0.01	0.25	0.38 ± 0.01	0.36 ± 0.01	0.12
EmC	0.28 ± 0.03	0.26 ± 0.02	0.22	0.32 ± 0.03	0.29 ± 0.01	0.34	0.35 ± 0.03	0.34 ± 0.01	0.79	0.39 ± 0.03	0.35 ± 0.01	0.14
EC	0.23 ± 0.02	0.23 ± 0.01	0.90	0.27 ± 0.03	0.26 ± 0.01	0.60	0.31 ± 0.02	0.29 ± 0.02	0.29	0.34 ± 0.03	0.29 ± 0.02	0.17
FX	0.24 ± 0.03	0.20 ± 0.02	0.21	0.27 ± 0.03	0.23 ± 0.02	0.45	0.29 ± 0.03	0.26 ± 0.02	0.15	0.33 ± 0.02	0.26 ± 0.02	0.04*
ILF	0.32 ± 0.03	0.29 ± 0.04	0.64	0.34 ± 0.03	0.33 ± 0.03	0.97	0.38 ± 0.02	0.35 ± 0.01	0.16	0.39 ± 0.03	0.35 ± 0.04	0.24
SLF Im	0.30 ± 0.02	0.31 ± 0.02	0.80	0.36 ± 0.02	0.33 ± 0.02	0.16	0.38 ± 0.03	0.35 ± 0.01	0.26	0.39 ± 0.01	0.35 ± 0.02	0.007*
SLF Ip	0.26 ± 0.04	0.23 ± 0.01	0.39	0.29 ± 0.04	0.25 ± 0.02	0.19	0.32 ± 0.03	0.27 ± 0.03	0.18	0.35 ± 0.04	0.31 ± 0.03	0.18
SLF IIa	0.42 ± 0.03	0.41 ± 0.02	0.50	0.46 ± 0.03	0.44 ± 0.02	0.35	0.49 ± 0.03	0.47 ± 0.01	0.42	0.53 ± 0.01	0.47 ± 0.02	0.03*
SLF IIm	0.44 ± 0.02	0.42 ± 0.04	0.55	0.47 ± 0.03	0.46 ± 0.05	0.81	0.50 ± 0.03	0.50 ± 0.03	0.83	0.52 ± 0.03	0.50 ± 0.04	0.66
SLF IIp	0.22 ± 0.02	0.21 ± 0.02	0.16	0.27 ± 0.04	0.25 ± 0.02	0.36	0.30 ± 0.02	0.27 ± 0.03	0.10	0.33 ± 0.04	0.29 ± 0.03	0.98
SLF III	0.33 ± 0.01	0.33 ± 0.03	0.08	0.38 ± 0.01	0.39 ± 0.02	0.90	0.41 ± 0.01	0.42 ± 0.01	0.31	0.45 ± 0.01	0.40 ± 0.03	0.002*

The data were tested with a two-way ANOVA and post-hoc analysis with significant level of p < 0.05 (*). SD: standard derivation. FA: fractional anisotropy.

**Table 4:** FA group difference at ages from 30 to 48 months.

Area	FA at 30 months (Mean ± SD)			FA at 36 months (Mean ± SD)			FA at 42 months (Mean ± SD)			FA at 48 months (Mean ± SD)		
	Control	HD	p	Control	HD	p	Control	HD	p	Control	HD	p
mPMC	0.41 ± 0.02	0.35 ± 0.02	0.01*	0.42 ± 0.02	0.34 ± 0.02	0.005*	0.41 ± 0.02	0.32 ± 0.01	0.007*	0.42 ± 0.03	0.30 ± 0.02	0.005*
VIP	0.44 ± 0.01	0.38 ± 0.01	0.004*	0.43 ± 0.01	0.36 ± 0.01	0.001*	0.42 ± 0.01	0.35 ± 0.01	0.001*	0.38 ± 0.02	0.32 ± 0.02	0.01*
TAa	0.32 ± 0.03	0.22 ± 0.05	0.006*	0.32 ± 0.03	0.21 ± 0.05	0.003*	0.31 ± 0.03	0.19 ± 0.04	0.004*	0.31 ± 0.03	0.18 ± 0.03	0.004*
MLF	0.43 ± 0.02	0.38 ± 0.01	0.02*	0.44 ± 0.02	0.38 ± 0.01	0.02*	0.45 ± 0.01	0.35 ± 0.03	0.001*	0.44 ± 0.01	0.33 ± 0.03	<0.001*
IC	0.43 ± 0.02	0.35 ± 0.01	0.01*	0.43 ± 0.03	0.32 ± 0.01	0.004*	0.43 ± 0.02	0.31 ± 0.03	0.003*	0.42 ± 0.02	0.29 ± 0.03	0.003*
AF	0.40 ± 0.02	0.35 ± 0.03	0.01*	0.41 ± 0.03	0.33 ± 0.03	0.01*	0.42 ± 0.03	0.31 ± 0.03	0.01*	0.40 ± 0.02	0.26 ± 0.04	<0.001*
CC	0.64 ± 0.05	0.57 ± 0.03	0.11	0.65 ± 0.06	0.58 ± 0.02	0.15	0.67 ± 0.04	0.58 ± 0.03	0.03*	0.68 ± 0.04	0.57 ± 0.04	0.01*
StB	0.40 ± 0.03	0.33 ± 0.02	0.04*	0.39 ± 0.02	0.30 ± 0.03	0.02*	0.38 ± 0.02	0.28 ± 0.02	0.006*	0.37 ± 0.02	0.25 ± 0.03	0.001*
UF	0.33 ± 0.02	0.29 ± 0.02	0.03*	0.34 ± 0.02	0.29 ± 0.02	0.004*	0.35 ± 0.02	0.27 ± 0.02	0.002*	0.35 ± 0.01	0.25 ± 0.02	0.001*
MdLF	0.37 ± 0.03	0.33 ± 0.03	0.15	0.38 ± 0.03	0.32 ± 0.04	0.07	0.39 ± 0.02	0.31 ± 0.04	0.004*	0.39 ± 0.02	0.29 ± 0.04	0.004*
CB	0.43 ± 0.02	0.35 ± 0.04	0.005*	0.44 ± 0.04	0.32 ± 0.02	0.02*	0.41 ± 0.02	0.30 ± 0.01	0.001*	0.40 ± 0.04	0.30 ± 0.02	0.02*
SS	0.32 ± 0.02	0.27 ± 0.03	0.02*	0.32 ± 0.02	0.26 ± 0.02	0.03*	0.32 ± 0.02	0.25 ± 0.04	0.002*	0.32 ± 0.02	0.24 ± 0.04	0.006*
dOB	0.39 ± 0.01	0.36 ± 0.02	0.03*	0.40 ± 0.01	0.35 ± 0.01	0.01*	0.40 ± 0.01	0.34 ± 0.01	0.004*	0.40 ± 0.01	0.32 ± 0.01	0.001*
EmC	0.43 ± 0.02	0.38 ± 0.01	0.03*	0.45 ± 0.02	0.37 ± 0.02	0.009*	0.46 ± 0.02	0.34 ± 0.03	0.008*	0.47 ± 0.02	0.33 ± 0.04	0.004*
EC	0.36 ± 0.01	0.27 ± 0.01	0.001*	0.35 ± 0.02	0.24 ± 0.01	0.004*	0.35 ± 0.03	0.22 ± 0.02	0.005*	0.33 ± 0.02	0.21 ± 0.01	0.003*
FX	0.35 ± 0.02	0.26 ± 0.01	0.001*	0.36 ± 0.02	0.24 ± 0.01	0.002*	0.34 ± 0.02	0.23 ± 0.01	0.003*	0.33 ± 0.02	0.23 ± 0.01	0.003*
ILF	0.40 ± 0.02	0.34 ± 0.03	0.06	0.40 ± 0.03	0.30 ± 0.01	0.007*	0.40 ± 0.02	0.29 ± 0.01	0.003*	0.39 ± 0.02	0.28 ± 0.02	0.004*
SLF Im	0.40 ± 0.02	0.33 ± 0.03	0.01*	0.41 ± 0.02	0.33 ± 0.04	0.007*	0.41 ± 0.02	0.33 ± 0.04	0.002*	0.41 ± 0.01	0.30 ± 0.03	<0.001*
SLF Ip	0.37 ± 0.03	0.31 ± 0.02	0.05	0.37 ± 0.02	0.28 ± 0.01	0.003*	0.36 ± 0.02	0.27 ± 0.02	0.004*	0.35 ± 0.03	0.26 ± 0.02	0.008*
SLF IIa	0.55 ± 0.01	0.47 ± 0.04	0.03*	0.56 ± 0.01	0.45 ± 0.03	0.003*	0.56 ± 0.01	0.43 ± 0.02	<0.001*	0.56 ± 0.02	0.42 ± 0.01	<0.001*
SLF IIm	0.54 ± 0.03	0.46 ± 0.02	0.045*	0.55 ± 0.02	0.45 ± 0.01	0.005*	0.55 ± 0.02	0.42 ± 0.01	0.002*	0.53 ± 0.03	0.41 ± 0.02	0.01*
SLF IIp	0.35 ± 0.03	0.31 ± 0.03	0.06	0.35 ± 0.03	0.28 ± 0.02	0.02*	0.35 ± 0.02	0.27 ± 0.02	0.007*	0.33 ± 0.02	0.24 ± 0.02	0.006*
SLF III	0.47 ± 0.02	0.39 ± 0.02	0.01*	0.48 ± 0.01	0.36 ± 0.03	<0.001*	0.47 ± 0.02	0.34 ± 0.03	0.001*	0.47 ± 0.02	0.32 ± 0.03	<0.001*

The data were tested with a two-way ANOVA and post-hoc analysis with significant level of p < 0.05 (*). SD: standard derivation. FA: fractional anisotropy.

## Discussion

White matter changes in developing HD monkey brains were investigated using DTI and TBSS analysis. Abnormal development patterns and micro-structural disruptions were observed not only in the fibers (i.e., StB and EC) connecting the cortical areas to the caudate and putamen, but also in multiple fiber tracts and few cortical areas across the whole brain. These findings are consistent with the widespread white matter loss reported in structural imaging in HD patients as the disease progresses^34^, and the different onset of white matter changes in various areas could reflect different functional alterations in progressive stages in HD monkeys^8^. The data suggest that assessment of white matter integrity could be an effective non-invasive method to evaluate disease progression in HD monkeys and to readily translate for patient diagnoses^35, 36^.

### Method aspects

The TBSS processing strategy offers voxelwise comparisons of DTI-derived measures on the skeleton maps of the whole brain to avoid inter-rater variability and has been widely used in DTI studies^32^. Temporal evolution of both brain maturation and aging has been analyzed with a quadratic regression model^37^. The quadratic regression model assumes even slopes across both the developing and degenerating periods, thus the trend of the change would not be realistic throughout the lifespan^38, 39^. In contrast, the Poisson model considers different slopes in developing and aging periods and was able to capture asymmetric changes in diffusivity measurements over the lifespan^37^. Evolution pattern of DTI indices on brain maturation and aging has been found to adequately follow the Poisson regression model in both human and non-human primates’ brains^15, 40^. As illustrated in Fig. 2, the DTI-derived measures in normal groups changed faster in the initial than the latter periods, suggesting that an increase in white matter (dendrites and spines) emerges quickly in early postnatal brain development but degenerate slowly as pruning of nonfunctional contact occurs, which is consistent with a previous study^40^.

As shown in Fig. 2 and Tables 1 and 2, significant age differences between controls and HD monkeys were detected for the minimum/maximum DTI-derive measures. The group differences for the maximum FA were detected in all areas, but those for the minimum MD and Dr were detected in fewer areas, such as CC and EC, whereas no group difference was detected for the minimum Da in any area. The results confirmed the general sensitivity of FA for the white matter changes in the HD model used. Although the mechanism is complex, FA changes in white matter could be due to alterations of myelination, axon size, fiber geometry and extracellular water space^21^. Although not as sensitive as FA, the complementary diffusivity indice changes in the white matter, such as Dr decrease, may reflect a disruption of the integrity of fiber myelin sheaths that was confirmed by electron microscopy^22, 41^. In addition, astrocytosis and/or microglia remodeling may change the diffusion anisotropy and diffusivity in the gray matter brain structures^23, 24^. Considering that gray matter contains large amount of microglia cells and neuronal cell bodies but small amount of myelin^42^, the FA changes in mPMC, VIP, and TAa could be due to either astrocytosis, neuronal remodeling, or loss of specific fiber tracts. In this study, the earlier emergence of the peak in white matter tracts maturation in the HD animals as compared to controls may mostly reflect an earlier demyelination process, whereas the earlier emergence of the peak in white matter in brain structures may be affected by earlier astrocytosis and/or microglia remodeling during development. The sensitivity of using FA was also demonstrated in the assessment of the time points at which the two groups differed in different areas (see Fig. 4 and in more details in Tables 3 and 4). The results also confirmed the ability of the Poisson regression model to detect group differences at specific ages, even though some minimum/maximum DTI-derived measures could not differentiate the two groups.

### White matter development in control monkey brains

Interestingly, in all cortical areas and fiber tracts (Fig. 4), control animals showed a sharp increase in white matter from birth until early adolescence (≈20–24 months) with sustained, yet reduced, growth thereafter through adolescence until adulthood (60 months). This developmental pattern parallels findings in an earlier DTI study in monkeys^43^. In addition, the peak of minimum/maximum DTI-derived measures follows a posterior-anterior pattern of the development with the posterior area reaching peak values slightly earlier than the anterior areas. For example, as seen in Table 1, the posterior parts of the superior longitudinal fasciculus (SLF Ip and SLF IIp) reached peak values earlier (38–39 months) than the more anterior parts (SLF Im: 47 months; SLF IIa: 57 months). Interestingly, the longest time to reach peak values was found in the most anterior frontal tract, the extreme capsule (EmC: 81 months). These data indicated a rough posterior-anterior trend for white matter development in these control animals, confirming the later white matter maturation in the frontal lobe and fiber connectivity already shown in human and rhesus monkeys^39, 43^. Furthermore, at 12 months of age, the FA value of frontal cortex fibers (e.g., EmC, SLF IIa) was within 0.32~0.46, whereas it was much lower (0.27~0.29) in more posterior fibers (e.g., 0.29 for SLF Ip and 0.27 for SLF IIp). These results were also consistent with previous results indicating that by 10 months (≈300 days) FA was highest in frontal regions but lowest in occipital regions^43^. All our results showed that the white matter development in the control animals parallels similar pattern of white matter development previously reported, though the development pattern might be slightly affected by the type of rearing conditions used in different studies.

### White matter alterations in HD monkey brains

In all areas analyzed, white matter in HD monkeys increased at roughly the same rate as controls from birth to early adolescence (≈24 months). However, as controls continue to show a slight white matter increase thereafter until adulthood, the HD monkeys demonstrated a significant decline. This development pattern suggests a widespread arrest, and then decline of white matter development in HD monkeys.

The hallmark of neuropathological changes in HD patients is the degeneration of caudate and putamen where atrophy takes place decades before the onset of motor and cognitive deficit^44, 45^. In HD monkeys, striatal growth ceased at 24 to 36 months of age, which is much earlier than that seen in control monkeys^8^. In the present study, maximal FA in StB fibers coursing in the external capsule on their way to the lateral putamen in HD monkeys occurred around 18 months of age, i.e. much earlier than that of the control monkeys (28 months). These data suggest early changes in the maturation of white matter that was detectable at least six months earlier than volumetric changes in the striatum of the same animal model^8^ and are also consistent with the progressive impairment in motor function beginning at 16 months of age in the same HD monkeys^8^. Thus, FA is a sensitive and reliable MRI-based marker for determining the alteration of the brain microstructural integration during HD progression.

In human, the caudate receives primarily projections from the lateral prefrontal cortical regions, whereas putamen receives projections from the motor areas (such as premotor, supplementary motor and primary motor cortices)^46, 47^. Changes of microstructural white matter of mPMC of HD monkeys was observed as early as 18 months of age, indicating that mPMC is one of the first cortical areas affected in HD model (Fig. 4a). These findings are in line with morphological data in human HD brains, indicating a loss of SMI32 immuno-positive pyramidal neurons in the primary motor cortex that may result in the loss of short projecting pyramidal neurons to the premotor area^48^. White matter alterations in HD monkeys were also found in fronto-striatal tracts, such as the StB and EC as well as dorsal occipital bundle (dOB) and may be associated with the decline of motor and visuo-motor functions shown earlier in the same animals^8^. These data are consistent with the visual cognitive decline reported in HD patients^49^.

Additional widespread white matter changes were observed in long association fiber pathways connecting the parietal lobe to the frontal lobe (SLF) and to the temporal lobe (MdLF and ILF), as well as in fiber tracts connecting the temporal lobe with the prefrontal cortex (EmC and UF) and the prestriate occipital areas with the temporal lobe, may be associated with the memory and cognitive decline observed in HD monkeys^50^, as well as in HD patients as shown by functional MRI study^51^.

Finally, abnormal white matter changes of the corpus callosum in HD monkeys indicate interhemispheric disruptions, which is also consistent with white matter microstructural changes and atrophy in corpus callosum in HD patients^52, 53^.

### Implications of the DTI findings in HD monkeys for HD in humans

To readily advance preclinical research for clinical translation, it is important to identify appropriate animal model with similar disease development patterns, to investigate pathogenic mechanisms and to evaluate effective treatment of inherited neurological disorders, such as HD^54, 55^. A non-human primate model is particularly important in modeling diseases such as HD because of progressive systemic changes throughout the body as individual aged. HD monkeys develop clinical features of HD progressively based on longitudinal assessment by neural imaging, cognitive behavioral assessment and molecular profiling studies^8, 17, 56, 57^. Through longitudinal DTI study, progressive spatial-temporal white matter changes in HD brains were revealed for the first time and aligned with symptom development as disease progresses in HD monkeys. Thus, HD monkeys can potentially be used as preclinical large animal model to facilitate the development of novel biomarkers through noninvasive imaging.

Compared to control subjects, this model shows widespread white matter alterations in brain areas (i.e., frontal, parietal, temporal, occipital lobes, cerebellum and brain stem) where macro-atrophy, micro-structural, and/or functional changes have also been found in HD patients^34, 58, 59^. Micro-structural white matter changes in the motor, sensory and cognitive brain areas are consistent with those observed in prodromal and symptomatic HD patients^58, 59^. However, longitudinal changes of white matter microstructures were rarely reported and these studies involved limited progressive disease stages and only adult HD patients^26, 30^. There were also case reports in juvenile onset HD patients, in whom volume losses were found in caudate nucleus and putamen^60–62^. These findings in juvenile onset HD patients are in agreement with the monkey results during brain development. However, due to the feasibility, developmental changes in white matter from infancy to adulthood have not yet been reported in human HD patients.

## Methods and Materials

### Animals

Three HD rhesus monkeys (*Macaca mulatta*, male) were generated by using lentiviral-mediated transgenesis as previously described^8, 63^. HD monkeys carry Exon 1–10 of the human *HTT* gene with expanded polyglutamine repeats (67Q, 70Q, and 72Q) under the regulation of the human HTT promoter^8, 57^. Four age-matched non-transgenic control monkeys (2 males and 2 females) were used in this study. During MRI scans, animals were anesthetized with 1–1.5% isoflurane mixed with 100% O_2_ and immobilized in a supine position in a custom-made head holder. Et-CO_2_, inhaled CO_2_, O_2_ saturation, blood pressure, heart rate, respiration rate and body temperature were monitored continuously and the body temperature was maintained with a warm blanket surrounding the animal. All procedures were approved and in compliance with the Institutional Animal Care and Use Committees (IACUC) of Emory University and the NIH guide for the care and use of laboratory animals. All animals were reared under the same conditions after birth. In brief, post-delivery, infants were surrogate/nursery-reared in the primate nursery of the Yerkes National Primate Research Center (Atlanta, GA) that allow normal growth as well as the development of species-specific social skills. All animals received the same treatments and procedures designed for the longitudinal MRI scans. They were monitored at least twice daily from birth by the research team or by animal care personnel at the YNPRC.

### Neuroimaging procedures

All MRI experiments were performed on a Siemens 3T Trio scanner (Siemens Medical Solutions USA, Inc., Malvern, PA). Diffusion images were acquired with a Siemens trans-receiving volume, 2-shot EPI sequence or a single-shot EPI sequence with GRAPPA acceleration (due to a scanner upgrade) with 8-channel phased-array volume coil and the following imaging parameters: TE = 89 ms, TR = 5700 ms, data matrix = 83 × 83, voxel size = 1.3 mm × 1.3 mm × 1.3 mm. DTI data were collected at b-value of 0 s/mm^2^ and a single b-value of 1000 s/mm^2^ with 30 diffusion encoding directions chosen to be approximately isotropically distributed on a sphere according to the electrostatic repulsion model. Whole brain field maps were acquired using a gradient echo sequence with TE = 6.24 and 8.7 ms, TR = 500 ms, FOV = 96 mm × 96 mm, voxel size = 1.3 mm × 1.3 mm, and slice thickness = 1.3 mm. T_1_-weighted images were acquired by using a 3D MPRAGE with the following parameters: inversion time = 950 ms, TE/TR = 3.5 ms/3000 ms, FOV = 96 mm × 96 mm, matrix = 192 × 192, 6 averages, and were used for structural identification and to construct an anatomical macaque template for the DTI image registration.

### Tract-based spatial statistics (TBSS) analysis

Data were processed with FSL (FMRIB, Oxford) and in-house MATLAB (Mathworks, Natick, MA) scripts. Voxel-wise TBSS analysis was derived using the TBSS toolbox in FSL (FMRIB, Oxford). FA maps were nonlinearly registered to a population-specific FA template, and then skeletonized (i.e., thinning non-maximal FA values perpendicular to the local tract structure) to produce a skeleton mean FA map that represents the major white matter tracts with reduced inter-subject variability. Then, each subject’s registered FA map was projected onto the skeleton by filling the skeleton with FA values from the nearest relevant tract center through searching the surface perpendicular to the local skeleton structure^32^.

The relationship between FA and animal age was examined by data fitting with a Poisson regression model using custom-written Matlab scripts (MathWorks, Natick, MA). Poisson model considers different change patterns during the brain development and degeneration as follows^40^:

1 $$FA=A\cdot age\cdot {e}^{-B\cdot age}+C$$

where A, B and C are the fitting parameters of the model.

The formula allows the ages at the maximal FA value to be calculated. After normal distribution of the calculated ages was confirmed by a one-sample Kolmogorov-Smirnov test, a two sample independent t-test was used to voxelwisely test the age difference within the skeletonized FA maps using false discovery rate (FDR) multiple comparisons correction with a q-value of 0.05.

In addition, mean diffusivity (MD), axial diffusivity (Da) and radial diffusivity (Dr) were also nonlinearly registered to a population-specific template averaged from the FA maps of all ages, and then skeletonized to produce the skeletons of the corresponding diffusivity maps. With the Poisson model described above, the age for the minimal diffusivities (MD, Da and Dr) were derived with the fitting parameters.

### ROI analysis

The brain regions with significant age differences in maximum FA between the controls and HD subjects were selected as regions of interest (ROIs; see Fig. 1). Localization of each ROI was performed using atlas of the rhesus monkey brain in stereotaxic coordinates^64^ and atlas of fiber pathways of the macaque brain^65^. Within each ROI, several other measures (the age at the maximum FA or minimal diffusivities, the maximum FA or minimal diffusivities, and FA or diffusivity values with increasing age) were averaged for further analysis (see Fig. 3). Independent t-tests were performed using FDR correction with q value of 0.05. Additionally, a two-way ANOVA with group as the between-subject factor and age as the within-subject factor followed by post hoc analysis with p < 0.05 as the significant threshold, was used to determine the FA difference between groups at different ages.
